# Successful Community-Based Conservation: The Story of Millbank and *Pterourus (Papilio) homerus*

**DOI:** 10.3390/insects8030069

**Published:** 2017-07-14

**Authors:** Eric Garraway, John Parnell, Delano S. Lewis

**Affiliations:** 1 Department of Life Sciences, University of the West Indies, Mona, Kingston 7, JMAAW15, Jamaica; eric.garraway@uwimona.edu.jm; 2 Department of Biology, Chemistry, and Environment Sciences, Northern Caribbean University, Mandeville JMDMR17, Jamaica

**Keywords:** Jamaica, giant, swallowtail, butterfly, ecotourism, indigenous, ecological knowledge

## Abstract

The literature on community-based environmental management is very extensive and the discussion of the pros and cons is continuing. Presented here is an example of a successful interaction between university-based entomologists and a local rural community, detailing the change in the attitude of the town of Millbank, Jamaica, from a Giant Swallowtail Butterfly collecting site to a model for community protection of a species and its environment. A review of some of the research work on community-based conservation efforts is included. These linkages take a considerable time to establish and the efforts spent by scientific personnel, governmental representatives and eco-tourists are itemized to emphasize how specific conservation activities have inspired confidence in the local community, thus engendering trust and mutual respect between the two groups. Reviews of the developed legislative support from both international and state entities also must be in place, and these are included in chronological detail as much as possible. Finally, a review of the long-term funding of educational and other local programs providing a level of stability to the conservation effort, until the local community can take over the protection of the species and/or habitat, is provided. Of utmost importance is a comprehensive educational campaign to not only sensitize the community, but the larger society, so that there can be buy-in from all stakeholders.

## 1. Introduction

Conservation success requires that local communities are engaged and shown how they can benefit from conservation efforts; this is the message that community-based conservation promotes [1]. Unfortunately, success is not always consistent or conclusive. Finding a good success story that is relatively free of controversy is of significant benefit to building a case for the use of community-based efforts in species or habitat conservation [2]. Paramount to conservation success of community-based efforts is a full understanding of the interactions between and among indigenous ecological knowledge, community-based conservation, and adaptive capacity in changing environments [3]. Mulrennan et al. [4] propose three core principles of community-based research to fix a perceived crisis that exists in this field. This crisis stems from the disappointing track record of conservation efforts, mainly resulting from: (i) over-simplified assumptions and misconceptions of “community”, (ii) the imposition of externally designed and driven projects, and (iii) a focus on conservation outcomes at the expense of community empowerment. These principles include a community-defined research agenda, a collaborative research process, and meaningful research outcomes [4]. When these principles are followed and conservation efforts take place as if the communities are also important to the conservation of a species or habitat, then success will be achieved. There needs to be a synergy between policy and practice in community-based conservation, where governmental agencies, non-governmental organizations (NGOs), and the local communities can come together. The present study highlights one such effort that we believe has enjoyed a level of success that could serve as a model for future use, and as a validation for the concept of community-based conservation.

*Pterourus (Palilio) homerus* is the largest member of the family Papilionidae; it is endemic to Jamaica and has long been sought after by collectors who are willing to pay significant sums of money for a specimen in good condition. It has always been a rare butterfly, and so the steady decline in numbers of sightings is most likely due to a combination of collection or poaching and, more recently, a significant modification of its habitat. Its geographic range is now limited to two locations: one in the Cockpit Country, an area of karst limestone formations containing strands of wet limestone forests in the center of the western end of the island (the western population); and the other in the Blue and John Crow Mountains, a set of peaks of volcanic origin located on the eastern end of the island (the eastern population) [5].

The small community of Millbank is nestled at the upper end of the Rio Grande Valley, sandwiched between the Blue Mountains and the John Crow Mountains. Initial visits by university entomologists to Millbank throughout 1984 eventually succeeded in establishing a working relationship with one of the local commercial butterfly collectors who was actively supplying international demand (mostly from the United States) for a variety of endemic butterfly species. At this time there were no specific Jamaican laws against collecting *P. homerus*, and butterfly collecting was regarded as a somewhat prestigious occupation in the Millbank community. In the same year, the concept of community-based conservation was first introduced to the local community by discussions with local leaders highlighting the importance of maintaining the populations of important endemic species. This concept was nurtured and developed throughout the following decades. Since the initial thrust by entomologists in the early 1980s through to the 1990s, visits in recent times to Millbank have been both irregular and varied. The occasional presence of researchers and eco-tourists has therefore become somewhat familiar to the local residents. In July 2010, 26 years after initial contact with the community, Eric Garraway was preparing to hike into the John Crow Mountains and was approached by two young girls, 10–12 years old, after they noticed the insect net he carried. They then proceeded to block the path and threatened to call the police because the researcher was “going to catch the butterfly”. The community-based conservation concept, which had been introduced many decades previously, had apparently matured and transcended the generations. The continuing, if not regular, contact between the scientific and the local community had evidently caused the residents of Millbank to become the official protectors of the species.

Research on the biology and ecology of *P. homerus* started as early as 1893, with records on its locality [6]. In 1939, the western population was discovered and this report was published in 1940 [7]. In 1985, an important legislative development occurred, with the inclusion of this species in the list of Threatened Swallowtail Butterflies of the World in the Red Data Book published by the International Union for the Conservation of Nature and Natural Resources [8]. In 1990, a comprehensive look at the ecology and conservation biology of *P. homerus* was completed [5]. This publication described the ecology of the remaining populations, and included detailed descriptions of the characteristics of the ideal habitat by delving into the altitudinal range, host plants, behavior, and other associated biological information of the species. Subsequently, additional research on parasitoid-induced egg mortalities [9], notes on the osmeteria [10], and additional contributions to the conservation of *P. homerus* were published [11,12]. In 2007, the population biology and ecology of the western population was published [13], and in 2008, additional new populations of the species were reported [14]. Since 2008, only two major articles have covered the ecology of *P. homerus*. The first stemmed from work done in 2008 [13], and reported on measuring wing damage of Lepidoptera [15]; the second looked at male-male interactions and discussed variables that possibly determined the outcome of territorial clashes, as well as lekking (an aggregation of males engaging in competitive displays), and the habitat that facilitated territory establishment [16]. Since then, research on *P. homerus* has not produced any scientific articles, and a comprehensive update on its ecology and conservation may be needed. This paper should not be considered as such, but as a case for a community-based conservation effort that makes much of the research possible.

## 2. Geographic Background

### 2.1. Millbank and Surrounding Areas

Millbank is a small, remote community, approximately 25 km inland from Port Antonio, the capital of the parish of Portland. The town is about 150 m above sea level and is nestled in the upper end of the Rio Grande Valley, sandwiched between the Blue Mountains and the John Crow Mountains. The John Crow Mountains reach elevations of up to 1000 meters and the Blue Mountains, 1300 meters. The slopes of the mountains are steep, especially the John Crow Mountains, and are characterized by numerous small ridges separating gorges, each gorge with its own stream (some temporary). Rainfall—mainly relief rainfall—is relatively high, as the mountains stand in the path of the Northeast trade winds [17]. The highest rainfall is at Corn Puss Gap, which receives over 7000 mm annually (30 year average), and the mean monthly rainfall for Millbank is 350 mm (40 year average; data from the Meteorological Office of Jamaica). This heavy rainfall promotes dense vegetation growth and an extremely high relative humidity, which favors the growth of the host plant and is ideal for the larval development of *P. homerus* [5,13].

In 1984, Millbank consisted of about 50 households and a population of approximately 150 individuals [18]. The whole area is predominantly a farming community; most persons do not own the lands on which they farm, but rather use Crown Lands (lands belonging to the state). Farms are generally small (0.5–1.0 ha), and farmers may shift from site to site, rotating over periods as short as two years, but some sites are permanent. Agricultural production was the dominant economic activity and employed 82% of the working population in 1998 [18]. The clearing of land was generally performed by the slash-and-burn technique, and most trees were generally removed; this practice puts it at odds with the characteristics of the habitat needed for the continued survival of *P. homerus*. However, a positive feature is provided by the geography of the area: much of the land slopes at an angle of over 25 degrees and is generally considered unsuitable for agriculture other than permanent tree crops.

### 2.2. Forest Industries Development Company (FIDCO)

The main threat of habitat loss did not come from subsistence farming from the surrounding community, but from the State. This started in the 1980s, with the emergence of the Forest Industries Development Company (FIDCO) established by the Government of Jamaica (1979). Its main aim was to significantly reduce the island’s importation of lumber by replacing the natural, forested areas with commercial pine plantations. Funding was provided by the World Bank and the Commonwealth Development Corporation. The target was to establish and manage 28,000 ha of commercial forests across Jamaica. The Government had substantial holdings of lands in the Rio Grande Valley: the upper areas were designated as Forest Reserve and were very strictly managed by the Forestry Department, and the lower regions were a mosaic of original forest, secondary forest in various stages of regrowth (some very mature), shifting cultivation, and pasture; these were made available to FIDCO.

The establishment of the commercial forests involved major infrastructural development, mainly in the form of new roads penetrating deep into the forests. The forest was clear-cut and replanted with a monoculture of *Pinus caribbea* seedlings. This left a huge environmental footprint; in comparison, the footprint of the community farms was miniscule. The arrival of FIDCO had significant impacts; many of the areas used by the farmers were now designated for pine plantations. Farmers were forced into new areas that were less accessible and sometimes less favorable for crops. This put further strain on the pristine habitat of *P. homerus*, and led to additional habitat loss at the hands of the community as they struggled to adapt. The newly planted pine plantations fared very badly before they became established, as the rate of growth of competing plants, especially vines, was much higher than FIDCO had encountered on the drier, southern slopes of the Blue Mountains, and the cost of managing this problem was far higher than budgeted. Moreover, there simply was not enough manpower to continue the expansion while caring for the young seedlings. Both planting and maintenance of the seedlings suffered badly, and the project soon ran into trouble. The need for far-reaching cultural shifts in work hours and habits by the community members employed on the project was also a limiting factor. Before the plantations could be fully established, disaster struck in 1989 in the form of Hurricane Gilbert, which devastated the pine plantations. The young pines broke like match sticks, and seedlings were washed away by flash floods and never regrew; thus began the decline of FIDCO. The pine forests were never replanted and the large expanses of hillsides were left naked; no reforestation programs were implemented. The farmers resumed their subsistence farming on selected plots, while much of the remainder was taken over by invasive species such as wild ginger (*Hedychium gardnerianum* and *Hedychium flavescens*) and common bamboo (*Bambusa vulgaris*).

FIDCO was based on a commercial philosophy, and work progressed so rapidly that there was no time to pay attention to the local customs or indigenous knowledge. An input from the community with an influx of indigenous knowledge could have prevented this blunder. For example, areas prone to landslides, which were cleared with disastrous consequences, could have been left alone if the indigenous knowledge of the community had been consulted and incorporated into the implementation and management plans.

## 3. Key Developments

### 3.1. The Years 1984–1991

#### 3.1.1. Commercial Butterfly Collecting

In 1984, there was a well-established local commercial butterfly collector in Millbank called Orlando Wilson (“the butterfly man”), who worked with anyone who was willing to purchase specimens of local butterfly species. A dealer based in the United States had established a working relationship with Orlando in which *P. homerus* was collected and sent, and Orlando was paid. Orlando had been supplied with butterfly-collecting equipment and had been taught how to package and post specimens to the United States. Besides this U.S. contact, other individuals occasionally visited in search of specimens. The best price Orlando received for a *P. homerus* specimen was US $50, but he generally received US $15–20. Collecting these butterflies was an attractive venture in Millbank at the time, as the daily wage for a farm-hand was about US $10. Moreover, this gave the collector a sense of prestige among the small subsistence farming community; fair money was made, and a collector could work and associate with “important” people. During this same period, a female specimen was advertised in the United States in 1984 for US $2800 [8] and US $3000. Information was also received on a second collector on the other side of the mountain, based at the town of Bath. This collector reportedly worked with several helpers and would remain in the forest around Corn Puss Gap for several days while on hunting trips. This time frame was understandable given that Corn Puss Gap was almost a day’s hike from the town of Bath. Efforts to locate or contact this collector were futile. Despite the presence of at least two main collectors, the major threat to the survival of *P. homerus* was considered to be the loss of suitable habitat.

#### 3.1.2. The 1984 Expeditions

The 1984 expeditions were conceptualized, organized, and partially funded by Dr. John Parnell, then a Senior Lecturer in the Zoology Department, Mona Campus of the University of the West Indies (UWI). Millbank was chosen for the expeditions as information had been received that *P. homerus* was actively being collected in the area, and secondly, little hiking was necessary to reach *P. homerus* habitat. Several trips were conducted from June to July, 1984, with the main aims being:

1. The establishment of a research base which allowed relatively easy access to the butterfly’s habitat.
2. The exchange of information with the local community.
3. The production of a short video on the species.

The establishment of a research base was deemed necessary because the typical access to *P. homerus* habitat through the town of Bath required four to five hours hiking; this left very little time for research on one-day exercises. The heavy rains also made access very difficult, and researchers often had to sit in tents and wait for several days for the butterflies to become active again after the heavy downpours. Millbank emerged as the ideal location as the town was nestled in the very foot of the forests, and *P. homerus* habitat could be reached in less than one hour’s hike.

The exchange of information with the Millbank community was considered essential; indigenous know-how and scientific knowledge were exchanged and the researchers gained an appreciation for the value of indigenous ecological understanding, such as ideal areas of maximum occurrence, the best times of the day and year for sightings, locations of local plants used as larval food-plants, and flowering plants preferred by adult insects. The huge amount of information that resided among the community that was passed down through the generations would have taken the most intense scientific project several years to unearth. Conversely, there was scientific information such as larval life histories, egg parasites, adult longevity, and other general environmental knowledge, to which researchers had access and that was shared with the local community.

The development of a video was considered vital to the success of this community-based conservation effort, as there was no other program aimed at disseminating visual information on the plight of the species. The video documented egg-laying, described the activity of both immature and mature larval stages on the food-plant, demonstrated pupation, showed the emergence of the adult, and discussed conservation problems. The ultimate goal of the video was to raise awareness both locally and internationally, hopefully resulting in legal protection and research. John Parnell left Jamaica in late 1984, but he had the video professionally edited by Ms. Carolyn Sides at the University Video Editing Center before he left. The Natural History Society of Jamaica (NHSJ) and the Department of Zoology, UWI, remained the main sources of support, including for the provision of opportunities for public engagements where the video was often shown, accompanied by slide shows and discussions. At the end of the 1984 expeditions it became clear that the species could not be protected without a community-based conservation effort, and three factors bore relevance to this realization:

1. There were no laws protecting the species from unlimited collection.
2. Even if there had been laws, the site was so remote that enforcement would have been extremely difficult without local support.
3. The culture of the community was closely intertwined with the forest, and this provided an ideal pathway towards the conservation of the species.

#### 3.1.3. Lacking Environmental Knowledge despite Vast Indigenous Ecological Knowledge

In 1984, the trade in *P. homerus* was unregulated, both locally and internationally. NGOs such as the NHSJ had lobbied the Jamaican legislature for the revision of the environmental laws and the establishment of a system of protected areas unsuccessfully for some years. The only protection was through a system of Forest Reserves. These reserves were well-respected by the community of Millbank; they considered it permissible to hunt wild pigs, harvest medicinal herbs, yams, and other forest resources from the reserve, but farming there was generally not practiced. Indigenous ecological knowledge in this community was vast, being grounded in the Maroon heritage [19]. There was a very intimate relationship with the natural environment, in which the community depended on the forests for various resources including hunting wild animals, food and drink, and medicinal and recreational herbs. Vivian Crawford (former Executive Director of the Institute of Jamaica) once explained that the maroons believe “there is an answer for every human ailment in the forest, and everything in the forest has a use.” They were in fact part of the ecosystem (understood a lot of its intricacies) and had a wealth of knowledge. In various ways, they understood many of the environmental factors that affected their agricultural yields [18,20].

Despite the vast indigenous ecological knowledge, an awareness of some key environmental issues was lacking. *P. homerus*, “the big butterfly”, was generally regarded by the community as very special, mainly because of its size and beauty. Also, because of interest showed by various visitors and the dollar value attached to each specimen, the butterfly became even more important to the community. However, there was no knowledge that it was endemic to Jamaica, or that it had a very limited distribution within Jamaica, and there was no concept of its conservation status. Conservation of *P. homerus* then seemed achievable if more information on the species was integrated into the culture of the community. A respect and understanding of community culture was essential, and mutual trust was necessary. Researchers learned from the indigenous ecological knowledge, while scientific expertise—not just on *P. homerus*, but also on general environmental information—was shared. Researcher-community interactions were recognized as a means towards both the empowerment of the community and the education of researchers.

#### 3.1.4. Developing Community Interactions

Field work continued with both one-day and extended visits to the Millbank area. Between 1985 and 1991, there were at least two one-day trips and one two-day trip per month. On the extended trips, entomologists were accommodated in the homes of members of the community, as the nearest guest house was over an hour’s difficult drive away in Port Antonio. This close interaction with the community was invaluable, as it allowed the development of trust as well as the opportunity to understand the local culture and learn from indigenous ecological knowledge, while sharing scientific knowledge. There were discussions on general environmental issues, and in particular, the plight of the butterfly. One primary aim was the conversion of Orlando Wilson from a butterfly collector to a data collector and conservationist. He was taught how to record basic field data, supplied with data sheets, and paid a regular stipend. A regular stipend ensured that he was at least as sound financially as when he received spasmodic payments for his butterfly shipments. The initiation of visits from educational or ecotourism groups was also an important part of the process, as the natural environment was a huge natural laboratory, and there was the added benefit of interactions between these groups and the community.

Using the Millbank Community Centre as a base made it possible to accommodate large groups. Annual visits by the NHSJ became a special event for the community, as such visits sometimes included town-hall meetings with open discussions on various environmental issues. It also became possible to conduct annual field trips for many undergraduate courses with students from UWI as well as the United States. Such field trips (at least three per year) allowed for a high level of interaction between visitors (students) and the community, with interactions of varying types; some visitors were even invited into the homes of residents in the community. Visits by groups, as well as field trips with students, had economic significance, as members of the community were employed as field guides, and visitors spent money directly in the bars, as well as purchased fruits, bamboo craft, cultural paraphernalia, and food from local shops. The potential impact (culturally and financially) may be appreciated when one considers 60 visitors embedded for periods of 2–7 days in a community of 50 households and 150 residents. These exercises helped to impart to the community that their natural environment was important, not just to them, but to the wider society. While these visits were conceived as educational rather than for tourism, they embodied most of the basic principles of ecotourism, such as building environmental and cultural awareness and respect, providing positive experiences for both visitors and hosts, and providing financial benefits and empowerment for the community.

#### 3.1.5. *P. homerus* as a Flagship Species

Due to the reliance of *P. homerus* on very specific environmental conditions of dense forest growth and high relative humidity [5,13], it was considered a prime candidate as a flagship species for habitat conservation in a broader context. By the late 1980s, *P. homerus* emerged as a flagship species for the conservation movement in Jamaica, and while it is yet to be declared officially as the national butterfly, it was treated as such; it has been used for several costumes in the National Independence Festival celebrations, and illustrations appear on souvenirs such as T-shirts and coffee mugs. Not surprisingly, when the Blue and John Crow Mountains National Park was finally established, the butterfly was chosen as its emblem. Later, there was a special issue of four Jamaican postage stamps in 1994, and it was placed on the most prized of phone cards of the Jamaica Telephone Company, as well as on the JAM $1000 bank note. Up until today, its status has not diminished, as *P. homerus* has become one of the key species in the present campaign to save the Cockpit Country (habitat of the western population) from commercial exploitation and environmental degradation.

#### 3.1.6. Legislative Protection

In January, 1988, just as the conservation efforts of *P. homerus* of the 1980s gained traction, Eric Garraway, along with fellow advocate David Hopwood, was requested to meet with the Hon. Anthony Johnson, then Minister of State in the Ministry of Agriculture, with a special responsibility for the environment. A briefing on the status of species was presented and the Minister made it abundantly clear that the government was in no position to stop the work of FIDCO, as the economic potential was simply too great for a struggling economy. Therefore, other means had to be investigated to save the species. There was need for amendments of existing legislation to afford protection for *P. homerus*, and a commitment was made towards the establishment of a system of protected areas, which would offer protection to the species contained therein and, in general, their habitat. Three main legislative developments initiated by the Minister subsequent to that meeting were:

1. Amendment of the Wildlife Protection Act to include *P. homerus* (gazetted in April, 1988).
2. Establishment of a system of protected areas (1990).
3. Major revision of laws related to the management of Jamaica’s natural environment. This resulted in the establishment of the Natural Resource Conservation Authority (NRCA) in 1991.

*P. homerus* was not protected under the Jamaican Wildlife Protection Act of 1945; this act focused mainly on terrestrial vertebrates and fishes. The first international protection was achieved under the Convention on International Trade in Endangered Species of Wild Flora and Fauna (CITES). A proposal by the United Kingdom in 1987 (CITES July 1987 Proposal No. CoP6 Pro. 56) resulted in *P. homerus* being placed on the CITES list which identifies species that are threatened with extinction. CITES prohibits international trade in specimens of these species, except when the purpose of the import is not commercial, for instance for scientific research. In these exceptional cases, trade may take place provided it is authorized by the granting of both an import permit and an export permit by the country of origin of the species.

Initial local official protection for this butterfly was provided by the Jamaican Wildlife Protection Act, which was amended in 1988 to include *P. homerus*. This Act is the only statute in Jamaica specifically designated to protect species of animals. Section 6 of the Act makes it an offence to have in possession any protected animal/bird or their parts and, under this Act, offenders may be fined or imprisoned for up to one year. Additional protection was provided by the Natural Resources Conservation Authority Act of 1991, which “provides for the management, conservation and protection of the natural resources of Jamaica”. Jamaica officially deposited an Instrument of Acceptance with CITES on 23 April 1997; this was accepted on 22 July 1997, and the island officially became one of the participants of CITES. The inclusion of *P. homerus* in the Jamaican Wildlife Protection Act and the official participation of Jamaica in CITES provided a completely new angle from which to approach its conservation.

Despite all the legislative successes for the conservation of this species and its habitat in general, there was no formal educational program to enlighten the populace, or even law enforcers of the new legislation. This is a shortcoming that, in hindsight, would have been pivotal for getting the message to the general populace, and possibly making the concept of a flagship species more understood by the rest of the Jamaican populace. Enforcing environmental laws is always very difficult, especially in developing countries, and a general educational campaign may have been helpful for preventing the emergence of new collectors. The fact that this butterfly often traversed areas utilized by the community members on their daily farming activities, and that the specimens were easy to hide, made enforcement difficult. The only guaranteed way to ensure adherence was through community-based education at the source, backed up by the dissemination of the knowledge that appropriate punishment of perpetrators would be imposed by the Government if violations could be proven in courts of law.

### 3.2. The Years 1991–2004

#### 3.2.1. The Rio Grande Research Station and Community Centre

Armed with new legislation and a sense of support from the state, a new research program began in 1990, which was the first long-term study of the species requiring researchers to remain in the field for extended periods. One of the main features was the establishment of a base, the Rio Grande Field Station, in 1991. The staff of the station consisted of a research assistant, Audette Bailey, a field assistant from the local community, Errol Francis, and Eric Garraway. The establishment of the field station allowed researchers to be more deeply embedded in the society, and involvement in the community took various forms. A small, informal school developed at the Rio Grande Field Station. Small children attended after normal school hours, and were engaged in classes that included basic academic materials (often school assignments), as well as fun and games, sometimes based on environmental material. Drs. Bailey and Garraway were invited to become members of a community group called the “Millbank Progressive League”. This group focused on community participation to tackle problems that arose. It also organized community activities such as “Ole Time Singting”, a festival celebrating the end of African slavery in Jamaica. It is important to note that the national celebration of the end of slavery was abolished in 1962, and was not re-established until 1997. The festival highlighted indigenous customs, especially those based on the use of natural products, or general relationships with the natural environment. The importance of conservation of local species such as *P. homerus* could easily be included in these celebrations. The preservation of this celebration by the Millbank community, as well as other communities across the island, was important to the re-establishment of these celebrations nationally.

The Rio Grande Field Station, and hence Millbank, emerged as focal points for research, teaching, conservation, and heritage efforts. The number of undergraduate visits, both local and international, increased significantly and included students from the United Kingdom. Several graduate projects were also initiated [18,21,22,23] and various researchers visited and worked in the area [24,25,26,27,28]. Visits by environmental NGOs, such as the NHSJ and the Jamaican Geographical Society, also increased. Because of the increased involvement of students, researchers, and tourists, several field guides were employed from the community on an “as required” basis, by both the field station as well as by visitors. These guides were educated on various key environmental and natural history issues, and two of these guides (Errol Francis and Donovan Grey) were also leaders in their community. These men played a vital role, and became ambassadors for the environmental movement. The Rio Grande Field Station closed its doors in 1999 due to a lack of funding, which led to a significant reduction in the number of visitors to the community; however, research continued through occasional visits. The loss of funding for the field station was a significant upset to the conservation efforts, and the community saw a decline in revenue from visitors as the frequency of visits decreased. Fortunately, the vacuum produced by the closure of the field station was soon filled by a community-based NGO.

#### 3.2.2. Blue and John Crow Mountain Nation Parks (BJCMNP)

The establishment of the Blue and John Crow Mountains National Park (BJCMNP) in 1991 provided a new avenue for both education and enforcement. The park covers over 48,000 ha and encompasses significant portions of the Blue Mountains and John Crow Mountains, including prime habitats for *P. homerus*. Moreover, as stated earlier, the park adopted *P. homerus* as its emblem. Millbank is situated in the buffer zone of the National Park, and the agricultural activities extend very close to the border of the park. There were occasional incursions into the park, but this was rare, as these areas were relatively remote. Generally, there was a great respect for what was formerly a Forest Reserve and what was then renamed a National Park. Owing to its proximity to the park, Millbank was immediately targeted by the park’s buffer zone management program. A residential ranger station, staffed by four park rangers, was established in the community, and these rangers, led by ranger Rudolph Poyser, played an active role in environmental education (informally, as well as though school visits), while at the same time acting as enforcers of the park regulations.

In 1993, the park launched a major environmental education program in conjunction with the Rare Center for Tropical Conservation (RARE), an NGO which had previously conducted environmental education programs in several eastern Caribbean countries. The RARE model utilized a threatened, endemic species of bird in each island as its focal point for the wider conservation message. However, the status of *P. homerus* and the availability of information on the species made it an ideal choice, and so the formula based on birds was amended [29]. This one-year program targeted school children primarily, and included songs, costumes, poems, posters, bumper stickers, broaches, and illustrated presentations. The BJCMNP suffered a significant reduction in budget throughout the early 1990s; the number of park rangers was reduced, and finally there were no rangers in residence by the last years of the decade. Although with a diminished presence, personnel of the National Park continued their involvement with the community. Several short education projects were organized, including one by The Nature Conservancy, which focused on the management of the freshwater systems of the area.

### 3.3. The Years 2004–2014

#### 3.3.1. Establishment of Bowden Pen Farmers Association (BPFA) and Ambasabeth Cabins

Driven mainly by demands to be organized as a group to capitalize on available markets for certain agricultural produce, the formation of the Bowden Pen Farmers Association (BPFA) in 2004 became a necessity. The former community group, the Millbank Progressive League, fused into this new organization. The new association was coordinated by community member Linnette Wilks, and organizational support came from the BJCMNP [19]. Environmental management soon became one of the aims of the BPFA. This was not surprising considering the level of awareness that then existed in the community due to previous efforts; a number of its leaders, members of the community, had been associated with the Rio Grande Field Station both as research field assistants and as tour guides; others had been associated with the BJCMNP in various capacities. This provided the core for the environmental movement, and not surprisingly, *P. homerus* remained the flagship species for habitat conservation in the area.

The BPFA soon established a set of cabins, the Ambasabeth Cabins, at Bowden Pen, and this became the hub for an ecotourism project [19]. The community had long been exposed to visitors and hence the BPFA had a base on which to build. Moreover, the high level of knowledge of biodiversity in the area, coupled with awareness of the environment in general, provided a good base for exchange between visitors and hosts [19]. The BPFA has since obtained a franchise from the BJCMNP to manage the two prime hiking trails in the area, the Corn Puss Gap Trail and the Cuna Cuna Pass Trail. These trails are now well-maintained, and the tour guides are fully certified. The guides are highly knowledgeable about the fauna and flora on the trails, especially the indigenous medicinal and culinary uses of the plants; the personnel at the cabins are all trained and certified [19]. The presence of the Ambassabeth Cabins as a physical base, and the logistic aid available from the associated personnel, has resulted in not only tourism, but the return of university courses, both local and foreign.

#### 3.3.2. Impact of the Bowden Pen Farmers Association (BPFA)

The impact of the BPFA on environmental management has been significant. The farming practices have changed: the use of fire to clear land has significantly diminished, practices to reduce erosion have been introduced (much less clear-felling), and *Hernandia* trees (larva food plant of *P. homerus*) are protected by mutual agreement. The group has been engaged in several re-afforestation projects. External funding was obtained [19], and members match these funds with voluntary labor. The trees selected for these projects were native, and special efforts were made to harvest seedlings from the surrounding forests. Emphasis was placed on including *Hernandia*, the larval food plant of *P. homerus*; this was important as a significant amount of this plant was removed by FIDCO in its clear-felling operations. The newly-planted *Hernandia* trees are now being utilized by the butterfly as breeding sites, increasing the possibility that visitors will see the butterfly, and thus improving the attractiveness of the tourism project. The activities of the BPFA thus include the encouragement of environmental best practices, as well as monitoring breaches of environmental laws. There is a strong informal education program passing the message to other members of the Millbank community, and to adjoining communities.

## 4. Conclusions and Recommendations

The story of the community of Millbank and *P. homerus* has emerged as perhaps the best example of ecotourism and community-based conservation in Jamaica, and one of the pillars of this success was the high level of environmental awareness in the community [19]. This concept of community-based conservation that was first planted in 1984 shows how positive relationships between researchers and communities can enhance conservation efforts, and this remains one of the few success stories that have since been documented and analyzed [30]. This concept is enduring and has transcended several decades and two generations in one community. In this case, the community has clearly become the protector of the species, and of the environment in general.

The presence of researchers, funding agencies, law enforcers, conservationists, and others is always finite, but the communities remain and continue to interact with the environment on an ongoing basis. Empowerment of the communities is essential in cases such as that described here. The development of mutual trust and understanding between researchers and communities is essential, but is very time consuming. Unfortunately, most researchers and administrators find it difficult to afford this time and rely on occasional visits; this has previously been discussed at length [31]. It is through the processes mentioned above that the ideas of researchers become part of the culture of a community, and indigenous knowledge adds to the research process; this gives the effort long-lasting impact, and a value that has clearly been documented [19].

Despite the successes enjoyed by this effort, several issues that work to the detriment or success of community-based conservation projects become evident. The first and most important issue is the need for a total immersion in the community. This is necessary if one is to gain the trust of the community members and learn from their indigenous knowledge. Researchers need to be totally immersed in the society for an extended period of time. This is often difficult, but is absolutely necessary for success. Secondly, and equally as important as the first, as with the building of any relationship, time spent is of upmost importance; as shown above, often time is spent not actually working directly on the conservation project, but on school assignments for community children, on town-hall meetings that may be looking at issues affecting farmers, and on dispute resolution among community members, to name a few, which are as important as working on the conservation of a species or habitat. It is the involvement of researchers in community-building activities that gains the trust of the members and softens their ears to the message the researchers bring; without these efforts, cooperation between groups would likely fail. Thirdly, state legislative and active support for the conservation effort makes up an essential part of the program. The requisite legislative framework must be established to add support to the community-based efforts. Without this support, there may be a level of success, however, this success will be short-lived if legislative protections do not prevent large agencies, including the state, from implementing wide-scale projects that undermine conservation efforts. Lastly, there is a need for long-term funding to support the sustained efforts, until the community is empowered enough to take over the protection of the species and/or habitat by themselves. This long-term funding should also take into account an educational campaign aimed not only at the community that is at the center of the conservation effort, but also to the larger community, so that there can be buy-in from the society at large. This would make legislators, teachers, practitioners, and all stakeholders aware of the issues, and the burden of conservation could slowly shift from just the local community to the society at large—making the community-based effort not just enduring, but endless.
